# Regression to the mean in latent change score models: an example involving breastfeeding and intelligence

**DOI:** 10.1186/s12887-022-03349-4

**Published:** 2022-05-16

**Authors:** Kimmo Sorjonen, Gustav Nilsonne, Michael Ingre, Bo Melin

**Affiliations:** 1 Department of Clinical Neuroscience, Karolinska Institutet, 171 77 Stockholm, Sweden; 2 Department of Psychology, Stockholm University, Stockholm, Sweden; 3 QUEST Center, Berlin Institute of Health at Charité – Universitätsmedizin Berlin, Berlin, Germany; 4 Department of Psychology, Linnaeus University, Växjö, Sweden; 5 Institute for Globally Distributed Open Research and Education (IGDORE), Stockholm, Sweden

**Keywords:** Analytical flexibility, Breastfeeding, Causal effect, Forward and backward change, Latent change score modeling, Maternal and child intelligence, Regression to the mean

## Abstract

**Background:**

Latent change score models are often used to study change over time in observational data. However, latent change score models may be susceptible to regression to the mean. Earlier observational studies have identified a positive association between breastfeeding and child intelligence, even when adjusting for maternal intelligence.

**Method:**

In the present study, we investigate regression to the mean in the case of breastfeeding and intelligence of children. We used latent change score modeling to analyze intergenerational change in intelligence, both from mothers to children and backward from children to mothers, in the 1979 National Longitudinal Survey of Youth (NLSY79) dataset (*N* = 6283).

**Results:**

When analyzing change from mothers to children, breastfeeding was found to have a positive association with intergenerational change in intelligence, whereas when analyzing backward change from children to mothers, a negative association was found.

**Conclusions:**

These discrepant findings highlight a hidden flexibility in the analytical space and call into question the reliability of earlier studies of breastfeeding and intelligence using observational data.

## Introduction

In this article, we use the effect of breastfeeding on intergenerational change in intelligence as a case study of regression to the mean in latent change score models. The effect of breastfeeding on intelligence is controversial. Earlier studies using observational data have found that breastfed children have higher intelligence compared to those not breastfed [1–4]. A risk for confounding is apparent, as breastfeeding mothers tend to be more intelligent than non-breastfeeding mothers or mothers who breastfeed only for a short period of time [4–6] and because intelligence is strongly hereditary [7–10]. A positive association between breastfeeding and child intelligence has indeed been shown to be less than completely attenuated when adjusting for maternal intelligence [2, 4, 5, 11]. However, since intelligence is measured with imperfect reliability, breastfeeding mothers may tend to have higher true intelligence than non-breastfeeding mothers even if they have the same measured intelligence. Hence, the remaining adjusted association between breastfeeding and child intelligence could be due to residual confounding. This example may illustrate the susceptibility of latent change score models to regression to the mean, a phenomenon which is well-understood in theory [12], but nevertheless often overlooked in practice.

Galton coined the term regression toward mediocrity to describe the phenomenon that tall parents tended to have tall offspring, but not quite as tall as themselves, while short parents tended to have short offspring, but not quite as short as themselves [13]. This phenomenon, nowadays usually called regression to the mean, occurs because extreme outcomes usually require an extreme combination of causative factors, and the probability is higher for some combination of causative factors that results in a less extreme outcome. So, even if an offspring has partly inherited their parent’s genome that increases the likelihood for tall/short stature, they may not experience the same extreme combination of other factors, such as nutrition, activity levels, and medical conditions, and this tends to result in a less extreme stature. An important feature of regression to the mean is that it has an effect backward as well as forward in time. Tall offspring can be expected to have tall parents, but not quite as tall as themselves, while short offspring can be expected to have short parents, but not quite as short as themselves.

The heights of parents and offspring in Galton’s example above are likely to have been measured with very high reliability. However, if we have an outcome Y that is measured with less than perfect reliability and a predictor X that has an association with the true value on Y, we can expect an association between X and observed change in Y between two measurements when adjusting for initial value on Y, even if no true change in Y has taken place. The reason for this spurious association is that with a positive (negative) association between X and the true value on Y, given the same initial value on Y those with a high value on X will tend to have a higher (lower) value on true Y and, consequently, a more positive (negative) residual in the measurement of Y compared with those with a lower value on X. And as residuals and measurement errors tend to regress toward a mean value of zero, those with a high value on X will tend to experience a more positive (negative) change in Y to a subsequent measurement compared with those with the same initial value on Y but with a lower value on X. The effect of X on the change score in Y is less susceptible to this fallacy when not adjusting for the initial value on Y [14–17].

Confounding refers to a phenomenon where two variables X and Y are associated without having any effect on each other because both of them are associated with a third variable Z. In attempting to evaluate whether X and Y are independently associated, it is common to estimate the association while adjusting for an indicator of Z. However, it is far from certain that such adjustment will eliminate the problem completely and some degree of residual confounding may remain. Residual confounding is increased by higher true degree of confounding, higher reliability in the measurements of X and Y, lower reliability in the measurement of Z, and larger sample size [18–22].

Latent change score modeling is a form of structural equation modeling for analyzing change in an outcome between measurements [23–25]. The use of latent change score modeling rather than traditional regression models has been recommended for analyzing change over time [24]. However, similarly to simpler regression models, latent change score models can be susceptible to the influence of regression to the mean if regressing the latent change score factor on the initial value on the outcome variable in addition to the predictor. For example, studies employing latent change score modeling have demonstrated what seems to be spurious effects of vocabulary on change in matrix reasoning scores, and vice versa, and of intelligence on change in academic achievement, and vice versa [26, 27]. Therefore, we have recommended to verify effects shown in latent change score models by analyses where the latent change score is not regressed on the initial value on the outcome variable [27].

Thus, we aimed to investigate the association between breastfeeding and child intelligence using two latent change score models susceptible to regression to the mean either on the mother’s or the child’s intelligence, in order to evaluate whether these approaches would diverge. If breastfeeding has a true causal effect on child intelligence, a positive association is predicted between breastfeeding and the latent intergenerational change score in intelligence, from mother to child, both when adjusting and when not adjusting for maternal intelligence. Moreover, a negative association is predicted between breastfeeding and backward intergenerational change in intelligence, from child to mother, when conditioning on child intelligence. This negative association would indicate that given the same intelligence, breastfed children tend to have mothers with lower intelligence and have, consequently, experienced a more positive intergenerational change in intelligence compared with non-breastfed children. Additionally, we simulated data with similar descriptive characteristics as in the empirical data and without any independent effect of breastfeeding on child intelligence, in order to test whether spurious associations would appear when it is known that no true effect is present.

## Method

### Participants

The present study employed data from a nationally representative sample of 6283 American female participants in the 1979 National Longitudinal Survey of Youth (NLSY79, available at https://www.nlsinfo.org/content/cohorts/nlsy79), born between 1957 and 1964, as well as data from the first (*N* = 4820) and second (*N* = 3328) born child of these women. We used this dataset because it is a large, openly available resource well suited to the question at hand.

### Measures

In 1980 a majority (*N* = 5939) of the women took the Armed Forces Qualification Test (AFQT). We transformed the score to an IQ scale (*M* = 100, *SD* = 15). Between 1986 and 2014 children aged five and over of the NLSY79 women could take the Peabody Individual Achievement Test (PIAT) in mathematics, reading recognition, and reading comprehension. The scores were normed to an IQ scale by the NLS personnel. At least one PIAT score was available for 3950 first and 2996 s born children, respectively. For those with more than one score available, a mean across the scores was calculated and used as a measure of their intelligence.

On nine possible occasions between 1983 and 1996 the NLSY79 women were asked if they had breastfed their first and second born child (when the child was an infant) at all. If they answered “yes” on at least one of these occasions and never answered “no”, the child was categorized to have been breastfed and if they answered “no” on at least one of these occasions the child was categorized to not have been breastfed. A dichotomous breastfeeding variable can be expected to be less susceptible to the effect of imprecise memory compared with reports of breastfeeding duration and, consequently, to be more reliable [28]. Data from 1983 on breastfeeding of subsequent children after the second were also available, but the cases were few (280, 62, 16, 2, and 1 for the third to the seventh child, respectively) and were not included in the present analyses.

### Statistical analyses

The association between breastfeeding and intergenerational change in intelligence was analyzed with latent change score modeling (see the Results section for illustrations). In one model, predicting forward intergenerational change in intelligence from mothers to children, child intelligence was regressed on maternal intelligence and a latent change score and both regression weights were fixed to one. The intercept and variance of maternal intelligence and the change score were freely estimated while they were fixed to zero for the child’s intelligence, i.e. the child’s intelligence was fully determined by maternal intelligence and the change score. The change score was regressed on maternal intelligence as well as on the dichotomous breastfeeding variable. Breastfeeding and maternal intelligence were allowed to correlate. In a second model, predicting backward intergenerational change in intelligence from children to mothers, maternal and the child’s intelligence changed places in the model. This second model was analyzed in order to distinguish between a true increasing effect of breastfeeding on child intelligence and a spurious effect due to regression to the mean. In a third model, predicting forward intergenerational change in intelligence from mothers to children, the regression effect between maternal intelligence and the latent intergenerational change in intelligence was replaced by a covariance.

As the aim was to compare models with different susceptibilities to regression to the mean, additional covariates were not included. For validation, models were run separately for first and second born children. Cases with missing values on all variables were omitted from the analyses, and for the rest missing values were handled by using full information maximum likelihood estimations. This resulted in sample sizes of *N* = 6172 and *N* = 6084 for the analyses involving the first and the second born child, respectively. Analyses were conducted with R 4.1.0 statistical software [29] employing the lavaan package [30]. Script and data are available at the Open Science Framework at https://osf.io/hnf8a/.

### Simulation

A dataset was generated through the following steps: (1) Two groups of virtual mothers were created, with the same sample size, mean true intelligence, and standard deviation of true intelligence as for the breastfeeding (first child) and non-breastfeeding mothers in the empirical data; (2) Each virtual mother was allocated a virtual child whose true intelligence correlated 0.8 with the mother’s true intelligence; (3) All mothers and children were allocated an observed intelligence score that correlated 0.8 with their true intelligence.

It is important to note that nothing in the data generation suggests an effect of breastfeeding on child intelligence over and above an effect due to a difference in maternal intelligence and heritability of intelligence. We used 0.8 as the population correlation between true maternal and true child intelligence and between true and observed intelligence as a reasonable approximation to correlations between observed maternal and observed child intelligence seen in the empirical data. Analyses with a correlation of 0.7 or 0.9 resulted in the same conclusion. The association between breastfeeding and intergenerational change in observed intelligence in the simulated data was analyzed with the same three latent change score models as for the empirical data (see above).

## Results

### Empirical analyses

Descriptive statistics for and correlations between the study variables are presented in Table 1. We see that 39% and 40% of the mothers breastfed their first and second child, respectively. We also see a positive correlation between breastfeeding and child intelligence, but a stronger positive correlation between breastfeeding and maternal intelligence.

**Table 1:** Descriptive statistics for and correlations between study variables

Variable	*N*	*M*	*SD*	Pearson correlation			
				2	3	4	5
1. IQ, mother	5939	100.00	15.00	0.506	0.518	0.385	0.393
2. IQ, child 1	3950	102.80	11.30	-	0.548	0.288	0.254
3. IQ, child 2	2996	101.73	11.31		-	0.276	0.270
4. BF^a^, child 1	4564	0.39	0.49			-	0.680
5. BF^a^, child 2	1771	0.40	0.49				-

^a^Breastfeeding, dichotomous variable; Note: All correlations are significant (*p* < 0.001)

**Fig. 1:**
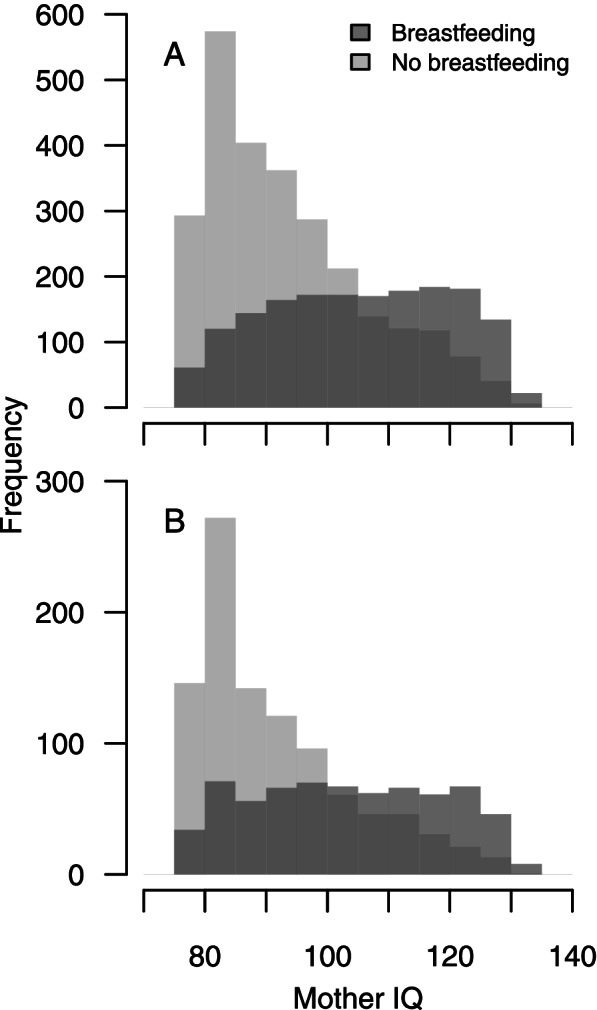
Maternal IQ frequency distribution, separately for first (A) and second (B) child and for those who breastfed (darker gray) or did not breastfeed (lighter gray) the child. Due to the scaling of the original variable (percentile from 0 to 100 with *M* = 42 and *SD* = 29), the range was restricted to 77.5–131

Non-breastfeeding mothers had lower measured intelligence than breastfeeding mothers (Fig. 1). Both for first- (panel A) and second-born (panel B) children we see that non-breastfeeding mothers were a rather homogenous group of low-performers with a peak at approximately IQ 85 (although with a positive tail reaching high scores as well). Contrarily, breastfeeding mothers were more uniformly distributed along the IQ-scale.

**Fig. 2:**
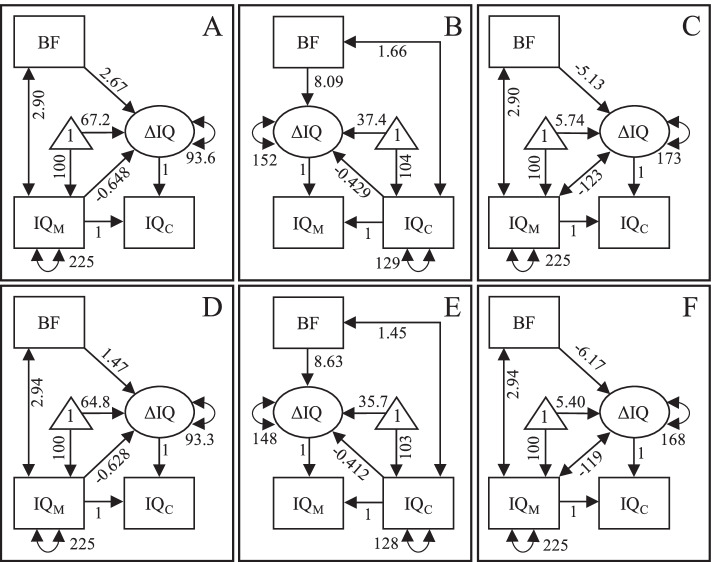
Models for predicting intergenerational change in intelligence from mother to child when conditioning on maternal intelligence (A and D), for predicting change backward in time from child to mother when conditioning on the child’s intelligence (B and E), and for predicting change forward in time from mother to child without conditioning on maternal intelligence (C and F). Separately for first (A-C) and second (D-F) child. Note: *BF* breastfeeding, *IQ*_*M*_ maternal IQ, *IQ*_*C*_ child’s IQ; the parameters are unstandardized; all parameters were statistically significant (*p* < 0.001, except for the effect of BF on ΔIQ in panel D, for which *p* = 0.012)

Predicted intergenerational changes in intelligence are presented in Fig. 2. For firstborn children (first row in Fig. 2) we see that: (1) If conditioning on maternal intelligence, breastfed children tended to have experienced a more positive intergenerational change in intelligence compared with non-breastfed children with equally intelligent mothers (panel A); (2) If predicting change backward in time from child to mother and conditioning on child intelligence, we still see a positive association between breastfeeding and intergenerational change in intelligence, meaning that breastfed children tended to have experienced a more negative intergenerational change in intelligence compared with equally intelligent but non-breastfed children (panel B); (3) If not conditioning on maternal intelligence, the intergenerational change in intelligence from mothers to children was predicted to have been more negative for breastfed compared with non-breastfed children (panel C). The results were similar for second-born children (panels D-F in Fig. 2).

The association between breastfeeding and intergenerational change in intelligence may seem very different in panels A and C in Fig. 2, with a positive and a negative effect, respectively. However, it should be noted that the positive effect in panel A ignores the positive association between breastfeeding and maternal intelligence. The expected (i.e. mean) IQ of mothers who breastfed their first child was 12.0 points higher compared with mothers who did not breastfeed their first child. If taking this difference into account, as well as the negative association between maternal intelligence and intergenerational change in IQ, the total effect of breastfeeding on the intergenerational change would equal 2.67 + 12.0 × -0.648 = -5.11, i.e. the same as the effect in panel C (difference due to rounding). Similarly, the expected difference in IQ between breastfed and non-breastfed firstborn children was 6.91 and if taking this into account the total effect of breastfeeding on backward intergenerational change in IQ, from child to mother (see panel B), would equal 8.09 + 6.91 × -0.429 = 5.13, which corresponds to the effect of -5.13 on forward change in panel C. The same logic applies to the effects on intergenerational change in intelligence from mother to her second child in row 2 in Fig. 2.

For cross-validation, we divided the full sample into two random subsamples (*N* = 3142 and *N* = 3141, respectively) and fitted the three latent change score models on data from these subsamples, separately for the first and the second child. As seen in Table 2, the effect of breastfeeding on intergenerational change in intelligence calculated in the subsamples resembled each other as well as the effects calculated in the full sample. This suggests that the effects are generalizable.

**Table 2:** The effect (with 95% CI) of breastfeeding on intergenerational change in intelligence in the full sample (*N* = 6283) as well as two random subsamples (*N* = 3142 and *N* = 3141, respectively). Separately for three alternative latent change score models (see Fig. 2 for illustration) as well as for first and second child

Child/Model	Full sample	Subsample 1	Subsample 2
Child 1			
Forward, Adj	2.67 (1.96; 3.37)	2.68 (1.67; 3.69)	2.65 (1.67; 3.64)
Backward, Adj	8.09 (7.23; 8.94)	8.16 (6.93; 9.38)	8.02 (6.83; 9.22)
Forward, Noadj	-5.13 (-5.98; -4.27)	-5.23 (-6.45; -4.01)	-5.02 (-6.23; -3.82)
Child 2			
Forward, Adj	1.47 (0.32; 2.62)	1.68 (0.15; 3.21)	1.23 (-0.49; 2.96)
Backward, Adj	8.63 (7.32; 9.94)	8.09 (6.24; 9.94)	9.12 (7.26; 11.0)
Forward, Noadj	-6.17 (-7.50; -4.83)	-5.88 (-7.70; -4.06)	-6.47 (-8.42; -4.51)

### Simulation

A simulated dataset with virtual breastfeeding (*N* = 1801, mean true intelligence = 103.3, *SD* of true intelligence = 15.1) and non-breastfeeding (*N* = 2763, mean true intelligence = 91.5, *SD* of true intelligence = 12.4) mothers was generated. Each virtual mother was allocated a virtual child whose true intelligence correlated 0.8 with true maternal intelligence. All mothers and children were allocated an observed intelligence score that correlated 0.8 with their true intelligence. Mirroring the empirical data, mean observed intelligence was set to 100 (*SD* = 15) across all virtual mothers and to 102.8 (*SD* = 11.3) across all virtual children.

**Fig. 3:**
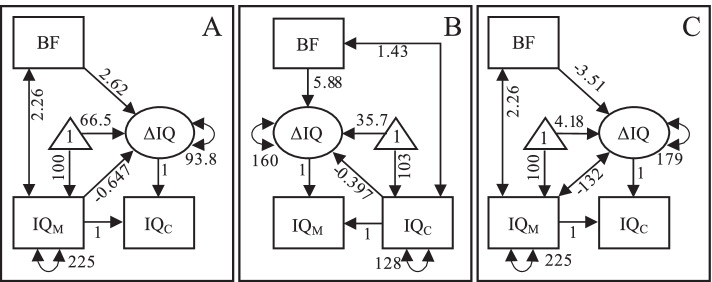
Findings in simulated data. Models for predicting intergenerational change in intelligence from mother to child when conditioning on maternal intelligence (**A**), for predicting change backward in time from child to mother when conditioning on the child’s intelligence (**B**), and for predicting change forward in time from mother to child without conditioning on maternal intelligence (**C**). Note: *BF* breastfeeding, *IQ*_*M*_ maternal IQ, *IQ*_*C*_ child’s IQ, the parameters are unstandardized; all parameters were statistically significant (*p* < 0.001)

Analyses with latent change score models of the simulated data yielded similar results as in the empirical analyses (compare Fig. 3 to the first row in Fig. 2). We see (1) a positive effect of breastfeeding on forward intergenerational change in intelligence, from mother to child, when adjusting for maternal intelligence (panel A); (2) a positive effect of breastfeeding on backward intergenerational change in intelligence, from child to mother, when adjusting for child intelligence (panel B); (3) a negative effect of breastfeeding on forward intergenerational change in intelligence when not adjusting for maternal intelligence (panel C).

## Discussion

We set out to evaluate if an observed association between breastfeeding and child intelligence differs between two methods of analysis with different susceptibility to regression to the mean either on the mother’s or the child’s intelligence. Consistent with earlier studies and with a true positive causal influence, a positive effect of 2.67 IQ points from breastfeeding on the intergenerational latent change in intelligence, from mothers to firstborn children, was observed when adjusting for maternal intelligence. However, contrary to a true positive causal effect, when adjusting for the first child’s intelligence, a positive effect of 8.09 IQ points from breastfeeding on the backward intergenerational change, from child to mother, was observed, meaning that breastfed children tended to have mothers with higher intelligence compared with equally intelligent but non-breastfed children and, consequently, to have experienced a more negative intergenerational change in intelligence. That the adjusted effect of breastfeeding on the intergenerational change in intelligence was positive both forward and backward in time suggests that it may have been due to regression to the mean. Also contrary to a true positive causal effect, the unadjusted intergenerational change in intelligence from mothers to children was more negative for breastfed children than for non-breastfed children. This latter negative effect should not be seen to definitively demonstrate a negative causal effect of breastfeeding on children’s intelligence. As many non-breastfeeding mothers had low measured intelligence, there was considerable scope for a positive, and limited scope for a negative, intergenerational change in intelligence from them to their children. Hence, the observed negative effect could be due to a floor effect. To be clear, we do not propose that breastfeeding has a negative effect on child intelligence.

The present results suggest that some of the observed positive effects in earlier studies, including aggregated effects in meta-analyses, may be due partly to residual confounding. Breastfeeding mothers may tend to have higher true intelligence than non-breastfeeding mothers with the same measured intelligence and this could be the reason why their children are predicted to have higher intelligence even when adjusting for measured maternal intelligence. Findings from the simulations in the present study indicated that a spurious positive effect of breastfeeding on child intelligence, or on the intergenerational change in intelligence, may emerge if (1) breastfeeding mothers are more intelligent than non-breastfeeding mothers; (2) intelligence is hereditary; and (3) intelligence is measured with less than perfect reliability. All three criteria appear to be established. Furthermore, it is possible that breastfed children tend to have more intelligent fathers, even when adjusting for maternal (true) intelligence. Intelligent fathers may be better at providing support – economic, emotional, etc. – that enables breastfeeding. Paternal intelligence is a possible confounder that has received very little attention in the literature.

It should be noted that there is no single proper way to conduct latent change score models, nor other statistical analyses, that results in infallible conclusions regarding causal effects. Difficulties to infer causality are inherent when analyzing observational data. As we did in the present study, researchers are recommended to analyze observational data with alternative models, e.g. to predict change both forward and backward in time. If interpretation of results from the models do not converge, findings may be spurious rather than indicating true causal effects. Stronger conclusions would be possible from observational data including repeated measurements of mothers’ and children’s IQ. Repeated measures would serve to increase the reliability of measurements of mothers’ IQ and to model a trajectory of children’s IQ, e.g. using growth curve analysis. Ideally, repeated measures of fathers’ IQ would also be adjusted for.

A few studies with stronger methodology than the ones mentioned so far have investigated the association between breastfeeding and child intelligence. Evenhouse and Reilly [31] compared the intelligence of breastfed and non-breastfed siblings, thereby completely adjusting for stable, e.g. genetically determined, maternal characteristics. They found a 1.68 percentile-points advantage for breastfed children compared with their non-breastfed siblings on an abbreviated version of the Peabody Picture Vocabulary Test, measured in adolescence. However, the breastfeeding or not of siblings was, of course, not randomized and it is possible that some child characteristics that may increase the likelihood for breastfeeding (e.g. ability to focus) are also positively associated with later measured intelligence, i.e. they act as confounders in the comparison of siblings. Der et al. [28] found no effect of breastfeeding on more comprehensive measures of child intelligence when comparing siblings in the same NLSY79 cohort as in the present study. Furthermore, Der et al. combined their results with those by Evenhouse and Reilly [31] and found the combined effect of breastfeeding on child intelligence to be weak and statistically non-significant. A cluster-randomized study found a significant effect of a breastfeeding promotion intervention on actual breastfeeding and on child verbal intelligence, at age 6.5 years, but not on child full-scale IQ [32]. However, it has been argued that the limited observed effect could be biased by the facts that (1) the study excluded mothers who had decided beforehand not to breastfeed their child, and (2) the pediatricians who conducted the measurement of intelligence were not blinded to the allocated condition of the child (the differences were smaller and statistically non-significant for blinded auditors who assessed a subgroup of the children) [6, 33]. Moreover, at age 16 no significant non-adjusted differences between the promotion and the control group remained, although a slight difference in verbal functioning could be observed if adjusting for various baseline characteristics [33].

Due to the limited, and possibly biased, findings in the randomized trial by Kramer et al. [32], the negative finding in the comparison of siblings by Der et al. [28], the risk of residual confounding in observational studies, even when adjusting for maternal intelligence, as well as the present findings, we think it remains premature to draw firm conclusions about a causal effect of breastfeeding on child intelligence.

The present findings illustrate that with the same scientific question regarding breastfeeding and intelligence, and in the same dataset, different analytical strategies can give strongly divergent results. Studies using a multi-analyst approach, i.e. where different analysts have tried to answer the same question using the same data, have identified other such divergences, for example concerning the effect of skin tone on receiving a red card in football [34] and the effect of decision making under risk on cerebral blood flow as assessed by functional magnetic resonance imaging (fMRI) [35]. The present findings thus provide an additional instance of an analytical space that permits a range of conclusions.

## Limitations

It is possible that the measurements of maternal and children’s intelligence used in the present study are not optimal, in regard to used instruments nor timing. It is also possible that there are several factors, i.e. maternal educational level and socioeconomic situation, home environment, etc., that may confound observed associations. Furthermore, the measurement of breastfeeding could have been a more nuanced measure of duration rather than a dichotomous yes/no-variable. However, it is important to bear in mind that such factors are constant across all three analyzed models (see Fig. 2) and cannot, consequently, explain the diametrically different estimates they provide. For example, as the data comes from the same mothers and children, maternal level of education cannot explain why the effect of breastfeeding on the intergenerational change in intelligence is positive in the model in panel A in Fig. 2 while the same effect is negative in the model in panel C. Hence, we consider ourselves entitled to conclude that estimates of the association between breastfeeding and child intelligence, or the intergenerational change in intelligence, are sensitive to the method of analysis. The full information maximum likelihood estimation assumes that missing observations were missing at random, which is not necessarily the case. The present study was conducted on a Western sample where the mothers were born between 1957 and 1964. Whether findings generalize to other times and populations is an open question.

## Conclusions

We have shown, using the effect of breastfeeding on intelligence as an example, that latent change score models are susceptible to regression to the mean, and that this phenomenon may lead to contradictory results. This finding calls into question the reliability of earlier studies of breastfeeding and intelligence using observational data. As studies of breastfeeding and intelligence using stronger designs have shown weak and inconsistent results, we conclude that a causal effect of breastfeeding on intelligence is far from established.

## Data Availability

The script and data are available at Open Science Framework at https://osf.io/hnf8a/.
